# A Structural Equation Model of Self-Regulation and Healthy Habits as an Individual Protective Tool in the Context of Epidemics–Evidence From COVID-19

**DOI:** 10.3389/fpsyg.2021.696813

**Published:** 2021-09-14

**Authors:** Sónia S. Sousa, Marisa M. Ferreira, Sara Cruz, Adriana Sampaio, Anabela Silva-Fernandes

**Affiliations:** ^1^Psychological Neuroscience Laboratory, CIPsi, School of Psychology, University of Minho, Braga, Portugal; ^2^The Psychology for Positive Development Research Center (CIPD), Lusíada University of Porto, Porto, Portugal

**Keywords:** COVID-19, self-regulation, healthy habits, mental health, well-being, structural equation model

## Abstract

**Objective:** The present study aims to explore the mediation role of self-regulation on health-related behaviors adoption or maintenance, mental health, and well-being during the COVID-19 confinement in a sample of adults in Portugal.

**Design:** One-hundred fifty individuals (118 females, 32 males; *M*age = 33.57 year; SD = 12.71) filled an online survey to assess self-regulation, healthy behaviors, mental health, and well-being perception, during the early months of the pandemic (June–August, 2020).

**Main Outcome Measures:** Self-regulation capacity, adoption or maintenance of healthy habits, mental health, including stress management, and the perception of one’s well-being were evaluated using a structural equation model (SEM).

**Results:** Self-regulation had direct effects on healthy habits and mental health and indirect effects on well-being and mental health mediated by healthy habits. In specific, a positive direct effect on healthy habits (β = 0.497, *p* < 0.001) and a negative direct effect on mental health (β = −0.428, *p* < 0.001); and a positive indirect effect on well-being perception, mediated by healthy behaviors and mental health (β = 0.253, *p* = 0.003), and a negative indirect effect on mental health, mediated by healthy habits (β = −0.208, *p* = 0.003). Additionally, healthy habits exerted direct effects on well-being perception and mental health. A positive direct effect on well-being perception (β = 0.254, *p* = 0.012), and a negative direct effect on mental health (β = −0.418, *p* < 0.001) were further observed. No direct effect of mental health was observed in well-being perception (β = −0.199, *p* = 0.068). Finally, a negative correlation was observed between self-regulation and weeks of confinement (*r* = −0.208, *p* = 0.021).

**Conclusion:** Self-regulation seems to be a good indicator of adopting a healthy lifestyle and better mental health and well-being in the context of the COVID-19 pandemic. Future preventive actions and interventions to build long-term global preparedness for future health emergencies, such as COVID-19, should explore the importance of self-regulation as an important individual and collective protective factor.

## Introduction

The world has faced pandemics in the past, the Spanish flu or the HIV pandemic, however, efforts to build long-term global preparedness for health emergencies seem to have been insufficient, considering the negative impact of the SARS-CoV-2 worldwide (WHO, 2021). The first coronavirus disease (COVID-19) case was diagnosed in a patient suffering from unknown pneumonia, in December 2019 at the Wuhan Jinyintan Hospital. A post-mortem histological examination showed bilateral diffuse alveolar damage of the lungs suggesting acute respiratory distress syndrome (WHO, 2020). As of 20 February 2020, the World Health Organization (WHO) described the SARS-CoV-2 as extremely contagious and capable of threatening many lives (WHO, 2020). To address the growing burden of COVID-19, governments and public health institutions in almost every continent adopted prophylactic measures, such as physical distancing and lockdown (e.g., Islam et al., 2020). While following these recommendations was and still is imperative to stop disease progression and for protecting lives, they also appear to have lead to profound changes in people’s lifestyle, mental health and well-being (Hossain et al., 2020; Rawat et al., 2021). Closed sports facilities, limited outing to buy food and increased psychological distress may negatively impact the ability to exercise and eating behaviors (Ammar et al., 2020; Hossain et al., 2020; Islam et al., 2020). Also, stressful life events, undoubtedly lead to psychological problems and hampers the quality of life (Hassanzadeh et al., 2017; Tibubos et al., 2020).

Recently, the scientific community has dedicated special attention in addressing the changes in lifestyle behaviors and the effects of the prolonged stays at home on mental health and well-being. A large body of available data nationwide suggests the containment measures have compromised physical activity levels. Reduced physical activity, mainly in self-reported moderate and vigorous physical activities and walking time, is documented in several countries, and across populations -adults and students- accompanied by an increase in sedentary time (Ammar et al., 2020; Castañeda-Babarro et al., 2020; Meyer et al., 2020). A change in the dietary behaviors was also largely evident, with studies reporting overeating, a higher consumption of fried and fast foods, and unhealthy snacks (Ammar et al., 2020; Ruiz-Roso et al., 2020; Rawat et al., 2021). Additionally, there is compelling evidence for low self-reported quality of life and increased psychological distress. Particularly, depression, anxiety, and stress-related disorders were highly reported by adults and students (Cao et al., 2020; Shovo et al., 2021; Brailovskaia et al., 2021; Chen et al., 2021). Evidently, the sudden episodes of obliged confinement to the home, the fear of the disease and the uncertainty about the future, among other factors, caused adverse mental health effects and disrupted people’s well-being (e.g., Brooks et al., 2020; Hossain et al., 2020; Tran et al., 2020). These changes are particularly worrying since insufficient physical activity and poor nutritional habits can compromise the immune system and infection susceptibility (Davison et al., 2016; Nieman and Wentz, 2019) besides the long-term consequences of these behaviors and the negative effects on physical and mental health.

A large body of research has investigated the role of self-regulation in the context of health treats and care (Leventhal et al., 1998; Graves and Carter, 2005; Janssen et al., 2013). This construct has gained popularity over the years and has been included into various models of Health Psychology. Self-regulation can be defined as the ability to develop, implement, and flexibly maintain planned behavior to pursuit one’s personal goals (Kanfer, 1970; de Ridder and de Wit, 2006). Individuals who are more self-regulated will sustain planned behavior over time despite failure, or adversity, to pursuit their goals (Oettingen et al., 2004). A plethora of evidence exists showing that self-regulation is an important mediator of numerous illness-related outcomes and health-related behaviors (Leventhal et al., 2016; Weidner et al., 2016; Elliston et al., 2017; Hagger et al., 2017). The adoption and maintenance of health-related behaviors in turn offer protection against physical and mental health issues (Locke et al., 2018; Briguglio et al., 2020). Overall, this previous work suggests that self-regulation is involved in health-related behaviors further supporting the notion that self-regulation may support individuals in maintaining healthy lifestyle behaviors and reporting more quality of life during a global pandemic.

Accordingly, the present study aims to explore the mediation role of self-regulation on health-related behaviors adoption/maintenance, mental health, and well-being during the COVID-19 confinement in a sample of adults in Portugal, using a structural equation model (SEM). We expect that individuals reporting higher self-regulation find it easier to adopt health-related behaviors, which in turn positively affects their mental health and well-being. Thus, the effects of healthy habits on mental health and well-being are mediated by self-regulation. Further, we expect self-regulation to have a direct effect on mental health and well-being. In a nutshell, it is hypothesized that individuals with more self-regulation engage more in health-related behaviors, report more well-being, and less mental issues.

## Method

### Study Design

**FIGURE 1:**
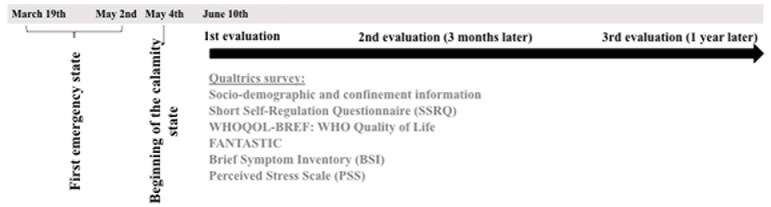
Study timeline.

Data was obtained as part of an ongoing longitudinal study investigating the impact of self-regulation in healthy habits, mental health, and well-being perception during the COVID-19 pandemic in Portugal. A cross-sectional design was adopted to analyze the baseline data in the early months of the pandemic (June–August, 2020) (see Figure 1).

### Participants

The target population were male and female adults aged 18 years or older (118 females; 32 males; mean age: 33.57 years ± 12.71) living in Portugal. Participants were recruited using a snowball method, via social media (Twitter, Facebook, Instagram, and Linkedin), media publications and promotions by institutions, University of Minho, Higher Institute of Health, Portuguese Psychologists board, and Foundation for Science and Technology. Followers on social media, and friends and colleagues were encouraged to share the survey. Informed consent was obtained via a cover letter explaining the study with the following statement at the end: “By clicking ‘I agree’ below, you acknowledge that you have read and understand the description provided, and as such consent to participating in this research study.” The study procedures were approved by the Ethics Committee for Research in Social and Human Sciences (CEICSH) of the University of Minho (approval CEICSH 052/2020) and followed the ethical principles for medical research involving human subjects of the World Medical Association (WMA) present in the Declaration of Helsinki (World Medical Association, 2013).

### Survey Details

Data in the present study was collected via an electronic survey during the early months of the pandemic when confinement was highest (June–August, 2020). An online platform (Qualtrics, XM, Provo, Utah) was used, being accessible by any device with an Internet connection. Data selected for the present study included five sections, namely demographics, the Short Self-Regulation Questionnaire (SSRQ), the Fantastic Lifestyle Assessment questionnaire (FANTASTIC), the Brief-Symptom Inventory-18 (BSI), the Perceived Stress Scale-10 (PSS), and the World Health Organization Quality of Life questionnaire (WHOQOL) – *Bref*.

### Measurements

#### Sociodemographic Information

Demographic variables included gender (*Gender*), age (*Age*), education (*Education*), and occupation (*Occupation*). Other variables of interest refer to items covering: the respondent or his 1st degree relatives are or have recently been infected by the SARS-CoV-2 (*SARS-CoV-2 infection*), the respondent is or has been in confinement (*Confinement*), working status during the confinement period (*Working status*), and financial status (*Financial status*).

#### Self-Regulation Assessment

Self-regulation was assessed with the Portuguese version of the SSRQ (Brown et al., 1999; Almeida and Behlau, 2017). SSRQ is composed by 31 items measured in a 5-point Likert scale (1 = totally disagree; 5 = totally agree), providing a total score and two subscores: (i) goal setting (*M* = 55.18; SD = 6.24; Min = 39; Max = 70) and (ii) impulse control (*M* = 56.15; SD = 8.33; Min = 33; Max = 73). Goal setting subscale assesses the ability to plan and set clear goals; and the impulse control subscale evaluates the ability to resist temptation, urges and impulses that may disrupt the goal directed behavior (Chen and Lin, 2018; Šebeňa et al., 2018). The total score ranges from 29 to 145 points reflecting self-regulation competencies, i.e., the ability to assess, plan, guide, and monitor a flexible behavior allowing the individual to adapt to the variety of environmental circumstances (Zimmerman, 2002). Higher scores indicate more self-regulation. Reliability analysis using Cronbach’s alpha coefficient revealed acceptable internal consistency for the goal setting subscale (α = 0.78) and good internal consistency for the impulse control subscale (α = 0.82; Gliem and Gliem, 2003). For statistics purpose were used the scale’s subscores.

#### Healthy Habits Assessment

Healthy behaviors were assessed using the *Fantastic Lifestyle Assessment* questionnaire (FANTASTIC; Wilson et al., 1984; Silva et al., 2014). Lifestyle refers to a set of habits and behaviors learned through socialization and constantly reinterpreted and tested along the life course in different social situations (DGS, 2004; Silva et al., 2014). The Fantastic questionnaire is composed by 30 items, scored between 0 and 2, addressing ten lifestyle components organized around physical, psychological and social domains: F, Family and friends; A, physical Activity/Associativism; N, Nutrition; T, Tobacco; A, Alcohol; S, Sleep/Stress; T, work/Type of personality; I, Introspection; C, health and sexual behaviors; O, Other behaviors. Each domain’s score is obtained by multiplying by 2 the sum of its items’ scores. The total score (0–120) is computed by summing all the domains’ scores. Higher scores reflect healthier habits and behaviors.

For statistics purpose was considered the total score (*M* = 89.49; SD = 12.18; Min = 44; Max = 118). Cronbach’s alpha coefficient revealed acceptable internal consistency for the total score (α = 0.77; Gliem and Gliem, 2003).

#### Mental Health Assessment

The presence of psychopathological symptoms was assessed using the BSI (Derogatis, 2002; Nazaré et al., 2017). The BSI is a self-report questionnaire used to identify depressive, anxious and/or somatoform symptomatology that may be clinically significant (Nazaré et al., 2017). It assesses the psychological distressed experienced by a person during the previous week to its completion, using a 5-point Likert scale (0 = not at all; 4 = extremely). From this questionnaire derives a total score, global severity index (GSI), and three subscales: somatization, depression, and anxiety. The GSI corresponds to the overall psychological distress level experienced, and the total score ranges from 0 to 72 points. The somatization subscale assesses distress symptoms related to autonomic system responses (e.g., gastrointestinal, cardiovascular); the depression subscale assesses core symptoms closely related to depressive conditions; and the anxiety subscale assesses symptoms related to panic states. Greater scores reflect more intense/severe psychological distress. For statistics purpose was considered the GSI score (*M* = 16.25; SD = 13.40; Min = 0.00; Max = 55.00). Cronbach’s alpha coefficient revealed excellent internal consistency for the GSI (α = 0.94; Gliem and Gliem, 2003).

Perceived stress was assessed with the PSS–*Portuguese Version* (Cohen et al., 1983; Trigo et al., 2010) comprising 10 items measured in a 5-point Likert scale (0 = never; 4 = very often). Perceived stress represents the extent to which life events are perceived as stress inducing as a result of their unpredictable, uncontrollable or excessive nature. Higher PSS scores reflect the perception of life events as more stress inducing. The total score ranges from 0 to 40 points and is obtained by summing all the items (*M* = 17.30; SD = 7.44; Min = 0; Max = 35). Cronbach’s alpha coefficient revealed good internal consistency for the total score (α = 0.88; Gliem and Gliem, 2003). For statistics purpose was included the total score.

#### Well-Being Assessment

Quality of life was assessed using the brief version of the WHOQOL, WHOQOL-*Bref* (The Whoqol Group, 1998; Vaz Serra et al., 2006). Quality of life represents “a person’s perception of his/her position in life within the context of the culture and value systems in which he/she lives and in relation to his/her goals, expectations, standards, and concerns” (WHO, 1994). It includes the “person’s physical health, psychological state, level of independence, social relationships, personal beliefs, and relationship to salient features of the environment” (WHO, 1994). The WHOQOL-*Bref* is composed by 26 questions (scored between 26 and 130 points), being the first two comprehensive questions in regards to one’s general perception of her/his quality of life and the general perception of health. The remaining questions assess the perception of one’s quality of life within four domains: physical, psychological, social relationships, and environment. Higher scores on WHOQOL-*Bref* represent greater well-being perception, either in general or in the specific domains evaluated. For statistics purpose were included the two initial questions of the questionnaire as they reflect one’s overall well-being perception (WHOQOL_BREF_O; *M* = 7.97; SD = 1.24; Min = 4; Max = 10). Cronbach’s alpha coefficient revealed poor internal consistency (α = 0.54; Gliem and Gliem, 2003).

### Statistical Analysis

#### Preliminary Analysis

Data was downloaded from Qualtrics and transferred to Microsoft Excel. Data was then scored and uploaded to SPSS Version 27.0. If there were any missing data points for any outcome variables, the participant’s entire data was removed from the analyses. Missing values for age (4 females), were treated as series of the mean using the mean value substitution method. All variables were evaluated for normality of distribution using a combination of histograms and the Kolmogorov–Smirnov test. From a total of the six variables included in the model, four of them were normally distributed (*p* > 0.05). Although the other two variables, BSI and well-being were not normally distributed, the normality was assumed. Well-being scores had histograms that looked normally distributed, while the BSI was positively skewed. To limit the effects of potential outliers, respondents who reported scores >3 standard deviations on either side of the mean for any of the variables reported in this study would be eliminated. No outliers were identified in the present study. Data was screened for the presence of psychiatric disorders. From a total of 158 participants, 8 of them were excluded because they reported being diagnosed with anxiety or depressive disorder. A total of 150 individuals with ages ranging between 18 and 68 years old (118 females and 32 males; mean age: 33.57 years ± 12.71) were included in the final sample. Descriptive statistics were computed for demographics, as well as for self-regulation, healthy habits, mental health, and well-being. Table 1 depicts participants’ detailed demographic information. Table 2 illustrates descriptive data for the remaining variables.

**TABLE 1:** Socio-demographic characteristics (*n* = 150).

	**Range**	***n* (%)**
**Gender** (Female/Male)		118 (78.67)/ 32 (21.33)
**Age** (Years)		
	18-68	150 (100)
	18-29	67 (44.67)
	30-39	37 (24.67)
	40-49	23 (15.33)
	50-59	17 (11.33)
	60-68	6 (4.00)
	Mean ± SD	33.57 ± 12.71
	Median	31.00
**Education**		
Middle school		2 (1.33)
High school		31 (20.67)
≥College		117 (78.00)
**Occupation**		
Professor		28 (18.67)
Researcher		20 (13.33)
Student		54 (36.00)
Health professional		17 (11.33)
Other		28 (18.67)
Unemployed		2 (1.33)
**Financial status**		
Very uncomfortable		3 (2.00)
Uncomfortable		5 (3.33)
Sufficient		69 (46.00)
Comfortable		61 (40.67)
Very comfortable		11 (7.33)
I prefer not to answer		1 (0.67)
**Self/household SARS-CoV-2 infection**		
Self		2 (1.33)
Household		7 (4.67)
**Confinement**		139 (92.67)
Weeks of confinement	Mean ± SD	8.85 ± 4.54
**Working status during confinement**		
Regular		2 (1.33)
Partial-time		8 (5.33)
Student -online classes-		55 (36.67)
Home Working		68 (45.33)
Lay-off		2 (1.33)
Unemployed		4 (2.67)

**TABLE 2:** Partial correlations controlled for age, gender and weeks of confinement.

**Variable**	***n***	**1**	**2**	**3**	**4**	**5**	**6**
1. SSRQ_G	150	–					
2. SSRQ_I		0.698***	–				
3. Healthy habits (FANTASTIC)		0.485***	0.387***	–			
4. Overall QOL and health (WHOQOL-BREF-O)		0.257**	0.266**	0.416***	–		
5. Mental health (BSI_GSI)		−0.375***	−0.460***	−0.599***	−0.359***	–	
6. Mental health (PSS)		−0.383***	−0.424***	−0.529***	−0.202*	0.690***	–

SSRQ_G, Short Self-Regulation Questionnaire – Goal Setting; SSRQ_I, Short Self-Regulation Questionnaire – Impulse Control; FANTASTIC, Fantastic Lifestyle Assessment; WHOQOL-BREF-O, World Health Organization Quality of Life – Brief version – Overall QOL and health; BSI-GSI, Brief Symptom Inventory – Global Severity Index; PSS, Perceived Stress Scale. *p < 0.05, **p < 0.01, ***p < 0.001.

#### Main Analysis

**FIGURE 2:**
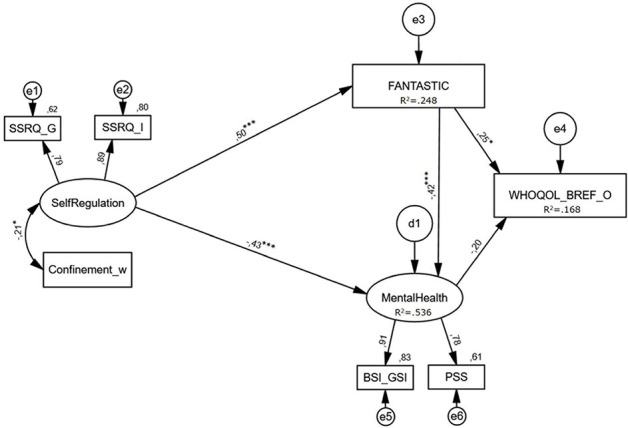
Results of Structural Equation Modeling Analysis. d, disturbance term; e, error term of each indicator; SSRQ, Short Self-Regulation Questionnaire (G, goal setting; I, impulse control); Confinement_w, confinement duration in weeks; FANTASTIC, Fantastic Lifestyle Assessment; WHOQOL-BREF-O, World Health Organization Quality of Life – Brief version – Overall QOL and health; BSI-GSI, Brief Symptom Inventory – Global Severity Index; PSS, Perceived Stress Scale; ns, not significant; *R*^2^, percentage of variance explained in each endogenous variable; *p < 0.05, **p < 0.01, ***p < 0.001.

A SEM analysis was performed using SPSS 27.0 (IBM Corp., Armonk, NY, United States) and AMOS 27.0., including self-regulation as exogenous variable; and healthy habits, mental health, and well-being perception, as endogenous variables. “Weeks of Confinement” was included as covariate (see Figure 2). SEM refers to a statistical technique that uses a combination of exploratory factor analysis and multiple regression, allowing for dealing with multiple variables, as well as testing hypotheses about how constructs are theoretically linked and the directionality of significant relationships. This method also allows evaluating how an “M” variable can mediate the relationship between two “X and Y” variables (Hox and Bechger, 1998; Schreiber et al., 2006; Bollen and Noble, 2011; Woody, 2011). The fit of the model was calculated based on the following multiple criteria: *X*^2^ test, goodness-of-fit index (GFI) ≥ 0.95, comparative-fit index (CFI) ≥ 0.90, normed fit index (NFI) ≥ 0.95, standardized root mean square residual (SRMR) < 0.08, and root mean square error of approximation (RMSEA) < 0.08 (Hooper et al., 2008; Kline, 2015). *Post hoc* power analysis was performed for each endogenous variable using the Free Statistics Calculators Version 4.0 (Soper, 2021). Hypotheses regarding the structural relationships of the constructs explored in the model were evaluated using the magnitude of path coefficients, standardized coefficient, and their significance. Bootstrap corrections with 500 iterations and 95% confidence interval were applied to the indirect effects (Byrne, 2010). Cronbach’s α for all the variables included in the model, and partial Pearson correlations (with bootstrap corrections, 5000 iterations and 95% confidence interval), controlled for gender, age, and confinement were computed. (Please see Tables 2, 3 for additional details).

**TABLE 3:** Descriptive Statistics and Cronbach’s alpha values for the variables included in the model.

**Latent variables**	**Observed variables**	**No of items**	**M ± SD**	**Cronbach’s α**
Self-regulation	Goal setting (SSRQ-G)	14	55.18 ± 6.24	0.78
	Impulse control (SSRQ-I)	15	56.15 ± 8.33	0.82
Healthy habits	FANTASTIC	30	89.49 ± 12.18	0.77
Overall QOL and health	WHOQOL-BREF-O	2	7.97 ± 1.24	0.54
Mental health	BSI-GSI	18	16.25 ± 13.40	0.94
	PSS	10	17.30 ± 7.44	0.88

*SSRQ_G, Short Self-Regulation Questionnaire – Goal Setting; SSRQ_I, Short Self-Regulation Questionnaire – Impulse Control; FANTASTIC, Fantastic Lifestyle Assessment; WHOQOL-BREF-O, World Health Organization Quality of Life – Brief version – Overall QOL and health; BSI-GSI, Brief Symptom Inventory – Global Severity Index; PSS, Perceived Stress Scale.*

## Results

Self-regulation had direct effects on healthy habits and mental health and indirect effects on well-being and mental health mediated by healthy habits. In specific, a positive direct effect on healthy habits (β = 0.497, *p* < 0.001) and a negative direct effect on mental health (β = −0.428, *p* < 0.001); and a positive indirect effect on well-being perception, mediated by healthy behaviors and mental health (β = 0.253, *p* = 0.003), and a negative indirect effect on mental health, mediated by healthy habits (β = −0.208, *p* = 0.003).

Additionally, healthy habits exerted direct effects on well-being perception and mental health. A positive direct effect on well-being perception (β = 0.254, *p* = 0.012), and a negative direct effect on mental health (β = −0.418, *p* < 0.001) were further observed. No direct effect of mental health was observed in well-being perception (β = −0.199, *p* = 0.068). Finally, a negative correlation was observed between self-regulation and weeks of confinement (*r* = −0.208, *p* = 0.021).

The fit of the model was met according to the following parameters: *X*^2^ (11) = 21.164, *p* = 0.032, SRMR = 0.043, RMSEA = 0.079, GFI = 0.962, NFI = 0.945, and CFI = 0.972. Based on *R*^2^ values, the final model accounted for 24.8% of the variance in healthy behaviors, 53.6% of the variance in mental health, and 16.8% of the variance in well-being perception. *Post hoc* power analysis were calculated for the three endogenous variables under study and revealed a higher power for healthy habits (99.99%), mental health (100.00%) and well-being (99.92%).

## Discussion

The present study sought to determine the relationship between self-regulatory skills, encompassed in goal setting and impulse control, and health-related behaviors, physical activity and preference for healthy foods, mental health and well-being, among adults in Portugal during the early lockdown stages of the COVID-19 pandemic (June–August, 2020). In line with our hypotheses, our main findings revealed that individuals with more self-regulation were engaged in more health-related behaviors, and reported better mental health and well-being during the early stages of confinement. These results highlight the role of self-regulation as a mediator of the individual’s adoption or maintenance of health-related behaviors, as discussed by authors in the field of health psychology (e.g., Hagger et al., 2017), emphasizing the protective role of healthy habits against mental burden (e.g., Briguglio et al., 2020). While we are unaware of any literature that examines the relationship between self-regulation and healthy habits adoption or maintenance during COVID-19, self-regulation has been associated with people being more physically active, consume more healthy foods, and report better mental health and well-being in non-pandemic times (De Bruin et al., 2012; Durand-Bush et al., 2015; Naughton et al., 2015).

Aligned with previous work, we found that highly self-regulated individuals reported better mental health and well-being, being this relationship mediated by healthy habits. In Hu et al. (2020) recent work during the COVID-19 outbreak in China, the authors observed that individuals with unhealthier lifestyle behaviors, such as reduced physical activity and decreased frequency of fruit, vegetables and breakfast intake, were more likely to report lower well-being, compared to those with healthier lifestyle behaviors. In a similar vein, Zhang et al. (2020) reported that the individuals who referred practicing exercise daily were more likely to exhibit better mental health, suggesting that practicing exercise daily may mitigate the negative effects of the COVID-19 pandemic on mental health. Our findings support Hu’s report emphasizing the effects of healthy habits on mental health and well-being during the COVID-19 pandemic. Regarding the relationship between self-regulation and mental health outcomes, in agreement to our findings, other studies highlighted the protective role of self-control -a subdomain of self-regulation- in mental health outcomes during the COVID-19 quarantine. Li et al. (2020) found that during the COVID-19 outbreak in China, individuals with higher self-control reported less mental health problems, compared to those with lower self-control. In a similar way, our data revealed that self-regulated individuals reported better mental health during the COVID-19 pandemic.

Another interesting finding that arose from our study was the negative association between self-regulation and weeks of confinement. Individuals who reported having passed more weeks in confinement were the one’s that also reported lower self-regulation. A potential explanation for this association is found in the Strength Model of Self-Control (Baumeister et al., 2007). The strength model proposes that the exertion of self-control, a subdomain of self-regulation, seems to depend on a limited resource. Behaviors requiring self-control, such as snack sugary foods when one knows that fruits and vegetables are better for the immune response or watch TV when should be exercising, cause short-term impairments -ego depletion- in self-control, leaving individuals more vulnerable to failure in self-control (Baumeister et al., 2007; Hagger et al., 2010). During the pandemic time individuals’ over-recruited self-control to maintain or adopt healthy behaviors to protect their own physical and mental health; while were dealing with the restrictions, the daily-live changes, and the deaths around the globe. All these might have contributed to increase the self-control burden, which will ultimately leave individuals more vulnerable to the negative effects of the pandemic. Although this theory might be thought-provoking our findings cannot support it as we did not analyze separately the subdomains of self-regulation. Future studies would be of interest to further address this hypothesis.

The present study has several limitations, including the cross-sectional nature of the data. However, this is an ongoing study and therefore more information about the consistency of health-related behaviors and mental health based on self-regulation will be learned through examination of the longitudinal data. Although the selected surveys are validated, there may have been some bias associated with the highly subjectivity and retrospective assessment of self-reported measures. Also, our sample was highly educated and therefore more self-regulation and healthier lifestyle behaviors may have been expected compared to the general public. Separate group analysis by age or occupation would also be of interest to understanding how the variables under study would relate to each other across different groups. While we have some students in our sample, we were not able to perform separate analysis due to the small sample size. Another drawback was the impossibility of analyzing separately the relationship between the subdomains of self-regulation -goal setting and impulse control- and the other variables under study -healthy habits, mental health and well-being. Due to the reduced sample size, the SEM analysis was performed with a reduced number of variables; otherwise we risked losing the model’s fit. Future studies should consider increasing the number of participants to confirm our results. Lastly, the COVID-19 pandemic is fast moving and physical distancing rules and confinement measures varied rapidly. These factors may have played a role in the self-regulation and health behaviors differences reported in this study.

Overall, the present findings suggest that self-regulation, encompassing goal setting and impulse control, may help individuals coping with adverse events such is the case of COVID-19, by actively engaging in health-related behaviors. Adopting or maintaining healthy behaviors, not only, but also during the COVID-19 pandemic is an urgent global health need. Harmful health behaviors may negatively affect the immune response leaving people more susceptible to be infected by the coronavirus and at risk for numerous chronic diseases at a longer term (Lange and Nakamura, 2020; Ng et al., 2020). Self-regulation has primarily been studied in the context of health treats and care. The Common-Sense Model of Self-Regulation (CSM), is a very popular model in this field and describes a multi-level process -perceptual, behavioral and cognitive- involved in individuals’ representations of threats to health, and procedures for self-management of ongoing and future health threats, by setting goals, creating action plans and implementing action for addressing the threat (Leventhal et al., 2016). The process is often initiated by deviations from normality (e.g., symptoms), by observation of illness in others, or from mediaand other environmental cues. These stimuli activate memories of the individual’s normal functioning self, past experiences of illness, treatments and lifestyle activities; and they generate mental representations of illness threats (i.e., cause, control, and consequences), possible treatments, and action plans (Leventhal et al., 2016). In a nutshell, according to this model, in face of a health treat such is the COVID-19, self-regulated individuals, will create and effectively implement a plan, such as for example, the adoption of an healthy lifestyle, in order to protect the self from the disease. The findings of our study seem to be aligned with this theory showing that more self-regulated individuals engaged more in healthy habits during the COVID-19 confinement, also reporting better mental health and well-being.

Finally, the present study has many strengths and the primary is related with the relevance of the construct -self-regulation- under evaluation, and its relationship with healthy habits, mental health and well-being, during such a life adversity as the COVID-19 pandemic. Also, to the best of our knowledge, this is the first study showing the role of self-regulation and healthy lifestyle behaviors in “protecting” people from mental disease in the face of a frightening illness such as the COVID-19. Thus, although acknowledging all the limitations, the findings derived from the present study seem an important contribution to the field of health psychology, since it provides important insights regarding individual differences in self-regulation that may predict the health and well-being of persons during extended confinement. This data can inform and contribute to the development of effective procedures of health promotion and serve as guidelines to design future preventive actions and interventions to face other pandemics in the future. As the COVID-19 pandemic is unfortunately still ongoing, our findings should be confirmed and investigated longitudinally with bigger samples to unravel if are lifestyle behaviors and mental health changing as the pandemic/confinement continues and what role does self-regulation play in these changes.

## Data Availability Statement

The raw data supporting the conclusions of this article will be made available by the authors, without undue reservation.

## Ethics Statement

The study procedures were approved by the Ethics Committee for Research in Social and Human Sciences (CEICSH) of the University of Minho (approval CEICSH 052/2020). The participants provided their informed consent to participate in this study.

## Author Contributions

SS wrote the manuscript, carried out subject’s recruitment and assessment, participated in the study design and data acquisition protocol, and carried out the statistical analysis. MF collaborated in subject’s recruitment and assessment, data collection, and manuscript writing. SC participated in the study design, carried out initial statistical analysis, and collaborated in manuscript writing. AS collaborated in statistical analysis and manuscript writing. AS-F participated in the study design, subject’s recruitment, assessment and data collection, carried out the statistical analysis, and collaborated in manuscript writing. All authors read and approved the final manuscript.

## Conflict of Interest

The authors declare that the research was conducted in the absence of any commercial or financial relationships that could be construed as a potential conflict of interest.

## Publisher’s Note

All claims expressed in this article are solely those of the authors and do not necessarily represent those of their affiliated organizations, or those of the publisher, the editors and the reviewers. Any product that may be evaluated in this article, or claim that may be made by its manufacturer, is not guaranteed or endorsed by the publisher.
